# No-tillage systems promote bacterial photosynthetic gene expression in low carbon, semi-arid surface soils

**DOI:** 10.1128/aem.00184-25

**Published:** 2025-03-10

**Authors:** Mark D. McDonald, Katie L. Lewis, Terry J. Gentry

**Affiliations:** 1 Department of Soil and Crop Sciences, Texas A&M University 199056 , College Station, Texas, USA; 2 Department of Natural Resources and the Environment, Center of Soil Biogeochemistry and Microbial Ecology, University of New Hampshire 3067 , Durham, New Hampshire, USA; 3 University of Louisiana at Lafayette, School of Geosciences 4365, Lafayette, Louisiana, USA; 4 Texas A&M AgriLife Research and Extension Center, Lubbock, Texas, USA; Washington University in St. Louis, St. Louis, Missouri, USA

**Keywords:** metatranscriptome, microbiology, soil, cover crops, no-tillage, semi-arid

## Abstract

Managing soils in semi-arid agricultural croplands generally focuses on reducing wind erosion, increasing fertility, and storing carbon. Thus, converting conventionally tilled systems to no-tillage and cover-cropped systems are often the first steps towards a conservation management approach across the growing area of semi-arid croplands. From a soil biological perspective, introducing cover crops to semi-arid soils has been shown to alter microbial community structure, which may lead to changes in the biogeochemical pathways expressed in these soils. In this study, we examined the impact of single-species wheat cover cropping and no-tillage on microbial gene expression after 4 and 5 years of implementation. We sequenced the metatranscriptomes of three production systems with varying levels of conservation management: conventional tillage winter fallow, no-tillage winter fallow, and no-tillage with a winter wheat cover crop. Removing tillage was the biggest factor altering microbial gene expression in this study, specifically resulting in upregulation of several photosystem-associated functions. These functions were taxonomically linked to organisms that make up the early stages of biological soil crusts, which may introduce additional benefits to these semi-arid agricultural systems beyond a reduction in wind erosion. Implementing a cover crop did not clearly alter gene expression beyond the effect of tillage removal; however, it did indicate a potential to reduce fungal disease incidence in 1 year of the study. These alterations of microbial activities and selection for potentially beneficial functions should be considered and further studied to aid in maintaining sustainable croplands for our changing climate.

**IMPORTANCE:**

Eliminating tillage from semi-arid agricultural soils has the potential to significantly alter the activities of the soil bacterial community compared with conventionally tilled soils. A major driver of this change was the activities of biological soil crust forming organisms that can provide several environmental benefits to the soil ecosystem beyond the typically associated benefits of conservation management. Furthermore, this study revealed that the implementation of a cover crop regime on no-tillage soils does not confer a major change in the function of the organisms present. Overall, the study reported here reveals that soil management practices aimed at reducing wind erosion and improving sustainability will positively impact the function of the microbial community and suggests that future investigations into the consequences of these functional changes may provide valuable services to these agricultural ecosystems.

## INTRODUCTION

Conservation management practices, such as no-tillage and cover cropping, have been widely suggested to improve soil health parameters (1). No-tillage refers to the removal of physical tillage of the soil for bed preparation and weed control. Cover cropping is often used in addition to no-tillage and is generally defined as the use of a secondary crop growth cycle during the typically fallow period of agricultural production, adding organic material to the soil. The addition of a cover crop to an agricultural production system can alter soil microbial communities and biogeochemical properties (2, 3). However, the strength of the impact these practices provide is often tempered by climate, environmental factors, and management of the native soil system (4–7). This is especially true for semi-arid systems, which are noted for having naturally low soil resources, such as soil carbon (C) and nitrogen (N), as well as below average microbial diversity and abundance (8). In these semi-arid soils, conservation management is most often implemented to reduce wind erosion and improve soil C resources (9).

Previously, we demonstrated that within 5 years of cover crop implementation, the microbial community is more consistent compared with those where cover crops were not used (10), although strong environmental control over the structure of the community was still apparent. However, changes in the microbial community do not necessarily reflect changes in microbial function (11, 12) or ecosystem outputs of the cover crop system, such as the lack of changes in soil C content after 19 years of conservation management in a similar semi-arid system (13). Prior analyses of this study also revealed a lack of change in ecosystem outputs (production of nitrous oxide, N_2_O) across the growing season (14) despite increased relative abundance of N cycling organisms (10).

In this study, we aim to determine what, if any, changes in microbial gene expression can be attributed to the implementation of conservation management practices that altered microbial community composition. To accomplish this, we conducted a metatranscriptomic analyses of three levels of conservation management: conventional tillage (CT), no-tillage winter fallow (NT), and no-tillage with a winter wheat cover crop (NTW). Metatranscriptomic analysis has been conducted in a variety of environments, including grassland and forest soils (11, 12), marine environments (15), and agricultural soils, where changes in microbial function under organic and conventional management were evaluated (16).

Previous determinations of microbial community structure in the soils referenced in this study (10) revealed a reduction in spatial and temporal heterogeneity (greater consistency in microbial community structure) under a cover cropping treatment (NTW). The reduced heterogeneity was attributed to the combination of management-induced changes in soil physicochemistry as well as the combined selection pressure of the cash and cover crops. With these results in mind, we hypothesized that reduced functional richness would be observed for the NTW system compared with a conventionally managed system (CT) or a no-tillage system without a cover crop (NT), which would be further observed as differences in the overall functional expression profile (beta-diversity of expressed functions). The reduction in richness would be related to a more consistent environment and community structure compared with the variability of the CT and NT systems, as was observed for microbial community structure. Furthermore, selection for functions within the “new” environment created by the NTW system would contribute to the observed difference in expression. Finally, we hypothesized that the observed changes in functional diversity will be largely related to differences in soil chemical and physical properties resulting from conservation system implementation, such as soil C content, gravimetric water content, and soil pH. In other words, we expect the NTW system to have fewer uniquely expressed functions due to reduced microbial diversity previously observed, and that the functions expressed would be fundamentally different than the CT or NT system due to the environmental change with cover cropping.

## MATERIALS AND METHODS

### Site description

This experiment was conducted on the semi-arid Southern High Plains at the Texas A&M AgriLife Research Center in Lubbock, Texas (33.687°, −101.827°), in a continuous cotton (*Gossypium hirsutum* L.) research trial. The objective of the research trial was to determine the impacts of soil conservation management system implementation on soil chemical, biological, and physical characteristics, as well as agronomic production shortly after implementation (<5 years). This study was conducted on a subsection of research plots previously described in McDonald et al. (17). The work here was conducted with a randomized complete block design with three replications where treatment was the level of soil conservation management (CT, NT, and NTW production systems). Nitrogen fertilizer was applied prior to cash-crop planting as urea–ammonium–nitrate (UAN, 32-0-0) at a rate of 168 kg ha^−1^. The rate of fertilizer was based on generalized fertilizer recommendations for this cropping area.

The cover crop in the NTW system was planted following cotton harvest and terminated prior to planting the following cotton growing season (cover grown November–May). Full details regarding the agronomic management of the field were published previously (17). Research plots were 15 m in length and covered four rows (1 m row spacing).

### Soil sampling

Soil at the research site is classified as an Acuff loam: fine-loamy, mixed, superactive, thermic Aridic Paleustoll (18). Soil samples were collected in August of 2019 and 2020 (4 and 5 years after conservation system implementation) to coincide with the timing of reproductive growth of the cotton cropping system. This time point represents an active time for plant growth as well as a combination of warm temperatures (mean temp Aug. 2019/2020 = 28.8°C) and higher soil moisture due to either recent precipitation (28 July 2020: 14.7 mm) or irrigation (furrow irrigation applied 30 July 2019) to promote plant growth. Four soil samples were collected within each plot roughly 10 cm from the base of the cotton plant row between rows 2 and 3. Samples were collected using 2.5 × 40 cm hand probes to a depth of 10 cm and homogenized. Soil samples were immediately (within 5 min of sampling) flash frozen using liquid N and stored under dry ice until transport to a −80°C freezer for storage until RNA extraction. In addition, six soil cores from each plot were collected, homogenized, and air dried for physicochemical analysis at both sampling points. Chemical analysis was conducted to determine soil mineralizable carbon via 3-day rewetting incubations with 40 g of soil (19), soil pH using a 1:2 soil–water dilution (20), nitrate (NO_3_—N), and ammonium (NH_4_^+^-N) were extracted using 1 M KCl (1:10 soil to extractant ratio, 4 g of soil) and analyzed for NO_3_^-^-N by reduction to NO_2_^−^ via cadmium followed by flow injection spectrometry (FIAlab 2600, FIAlab Instruments Inc., Bellevue, WA) and for NH_4_^+^-N by the Berthelot reaction involving salicylate (21). Gravimetric water content (GWC) was determined for each sample by drying soils for 7 days at 60°C.

### RNA extraction

Total soil RNA was extracted from each homogenized sample using a Qiagen RNeasy PowerSoil Total RNA kit with slight modifications to increase RNA yield and decrease organic contamination. Specifically, solution IRS volume was increased to 1 mL, and solution S3 was added at a volume of 2 mL (Qiagen LLC USA, Germantown, MD). Immediately following RNA extraction, eluted RNA was purified using a RNeasy PowerClean Pro CleanUP kit following manufacturer protocol, with a final elution volume of 75 µL (Qiagen LLC USA, Germantown, MD). Samples were then stored at −20°C until analysis could be conducted. In addition, initial soil input was increased for several samples to aid in RNA extraction yield where samples were lysed individually and recombined into a single extraction at the first filtering stage.

### Library construction and sequencing

Library preparation and sequencing were conducted at the Texas A&M Institute for Genome Science and Society. Ribosomal RNA depletion was not conducted for this experiment due to low RNA extraction yields. The low RNA extraction yields reflect the lower level of microbial activity present in these semi-arid soils and may reduce the comparability of the findings here to studies where microbial activity, and thus gene expression, is greater. Extracted RNA was quantified and evaluated for degradation with an Agilent Technologies 2200 TapeStation using the High Sensitivity RNA ScreenTape System protocol with 2 µL of RNA extract (Agilent Technologies Inc., CA, USA). Library preparation for RNAseq was conducted according to the TruSeq Stranded Total RNA protocol (Illumina Inc., CA, USA) with slight modification. Library preparation began with adding 8.5 µL of extracted RNA to the depleted RNA fragmentation plate (DFP) with no prior cleaning steps. No control was used for the library preparation, and resuspension buffer (RSB) was substituted where indicated. The final library was quantified with a Qubit 2.0 Fluorometer (ThermoFisher Scientific Inc., MA, USA) and with an Agilent 2200 TapeStation for bp size information. The nM concentration was calculated, and samples were normalized in a two-step process. Samples were first normalized to 10 nM, followed by normalization to 4 nM in low EDTA. Final 4 nM samples were pooled and sent to the North Texas Genome Center for sequencing with an Illumina NovaSeq 6000.

### Transcript sequence processing

Initial bioinformatic analysis was conducted using the GRACE cluster maintained by Texas A&M High Performance Research Computing. Raw sequences were evaluated for quality using the FASTQC tool (22). Reads were trimmed using AdaptorRemoval v2 (23). Locally, rRNA transcripts were removed using ribodetector (24). Each sample’s paired read files were then further processed using the online Metatranscriptomics Workflow (v0.0.3) from NMDC EDGE (https://nmdc-edge.org/metat/workflow). Briefly, the workflow checked samples for rRNA using the SILVA rRNA database, assembled transcripts using MEGAHIT, and annotated the assembled contigs using the Metagenome Annotation Workflow (v1.0.0). Annotation is conducted in two steps in this workflow. Contigs are first structurally annotated using tRNAscan_se, RFAM, DRT, Prodigal, and GeneMarkS. The results of these structural annotations are merged to create a consensus from which the functional annotation is generated. Functional annotation through the Metagenome Annotation Workflow was conducted using several protein family databases (SMART, COG, TIGRFAM, SUPERFAMILY, Pfam, and Cath-FunFam) to generate consensus annotations where applicable.

Following the generation of contigs and contig annotation in NMDC EDGE, these files were combined across all samples to create a generalized study-wide database of annotated contigs to improve transcript identification and quantification. Redundant contigs were removed from the study-wide database using cdHIT (25). Following redundancy correction, transcript abundance was quantified using Kallisto (26), where the study-wide database was used to generate the index for quantification and the rRNA-removed forward and reverse reads were used as the input.

The estimated count outputs from Kallisto were then used as the input to evaluate differential expression using DESeq2 (27) in R (v4.3.2) (28). Two different approaches were used to analyze differential expression. First, the “raw” estimated count of each contig was analyzed to represent transcript-level expression differences. This transcript-level analysis is presented in the supplemental material and represents a more inclusive view of gene expression changes that accounts for differences between which microbes are conducting which functions. Second, the overall differential expression of functions was conducted to identify any community-wide changes in functional abundance and expression. To accomplish this, contigs with identical annotations were identified, and the estimated counts were summed to generate a matrix of the total number of transcripts mapping to each functional annotation. To determine identical annotations, we first examined the total annotation generated by the NMDC pipeline and determined the most abundant annotation source, and then hierarchically assigned a single annotation for each contig following the pattern: if KEGG present, then annotate as KEGG, followed by COG, Pfam, SUPERFAMILY, and TIGRFAM. We chose to highlight this second approach in the main text due to the large amount of differentially expressed transcripts that mapped to similar or identical functions during the first transcript-level analyses. Furthermore, we chose to present these findings in the main text as they represent a more conservative approach to observing and reporting potential functional changes due to the implemented soil management strategies.

### Statistical analysis

Statistical analyses for soil physicochemical parameters were conducted in R (v4.3.2). Analysis of variance was conducted with a repeated measures approach for soil parameters and abundance of unique transcripts in each sample using the lmer function in the lme4 package (29). Fisher’s protected LSD was used to separate means of significant effects at α = 0.05 with the lsmeans package (30). Differential expression analysis including principal components analysis and gene clustering using regularized-logarithmic (rlog) transformation of transcript quantities and Euclidean distance was conducted within the DESeq2 package in R (27) for both transcript- and gene-level expression as well as within years. Correlation of soil chemical parameters to principal components was conducted using the rcorr package (31). PERMANOVA of expression distance matrices was conducted using the adonis2 function in the vegan package (32) and pairwise comparisons were conducted using the pairwise.adonis function from the pairwiseAdonis package (33).

Normalized functional counts were extracted from the DESeq2 analyses and used to generate a phyloseq (34) object to visualize relative abundance differences between production systems across years. Only the top 50 most abundant functional annotations were included, and all remaining functions were summed for the visualization. Visualization of the PCAs generated from DESeq2 and relative abundance were conducted using ggplot2 (35). Volcano plots of differentially expressed functions were generated using the EnhancedVolcano (36).

## RESULTS

### Soil characteristics

**Fig 1:**
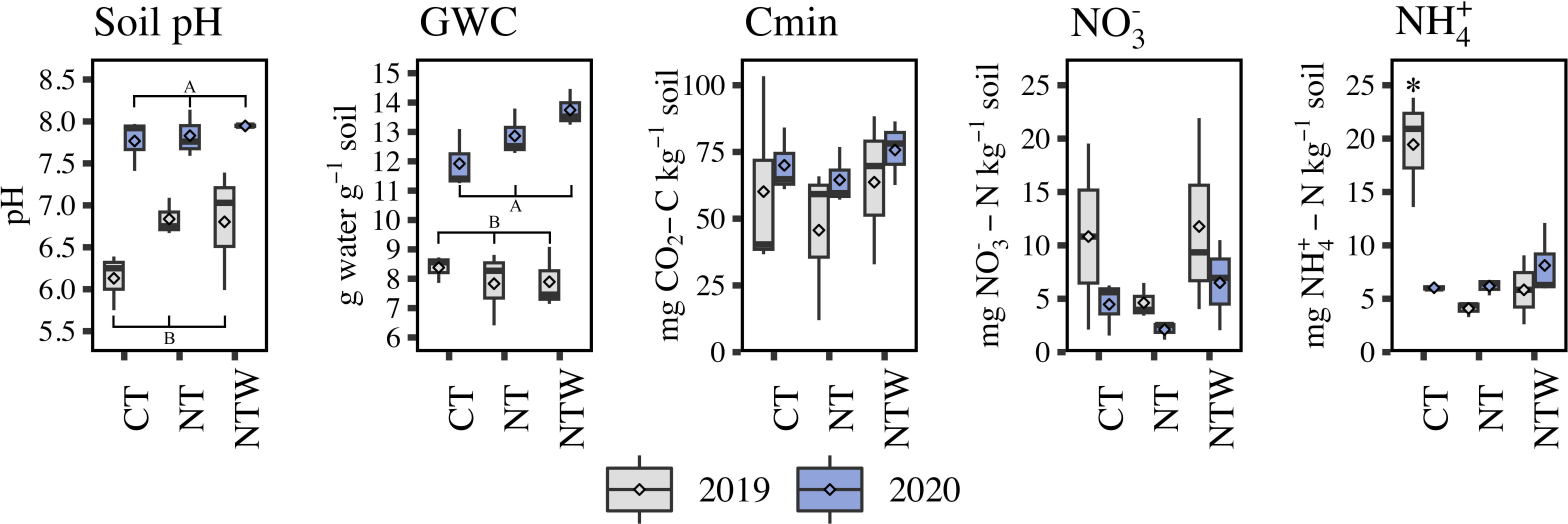
Soil characteristics by production system and year. GWC, gravimetric water content; Cmin, mineralizable carbon content; NO_3_^−^, nitrate-N concentration; NH_4_^+^, ammonium-N concentration. LSD letters represent yearly differences in soil pH and GWC at *P* < 0.05. The starred point in the NH_4_^+^ analysis (CT 2019) is different from all other production system*year interactions at *P* < 0.05. Boxes extend from the 25th to 75th percentile, where the central line indicates the median. Diamond shapes within each box represent the mean, and whiskers represent the inter-quartile range from the 25th to 50th percentile.

Soil NO_3_^-^-N and Cmin were unaffected between years and were not different between production systems (Fig. 1; Tables S2 and S3). However, soil pH and GWC were different between years, where both parameters were greater in 2020 than in 2019 (*P* < 0.001, Fig. 1). Because of the strong year-effect on soil pH and GWC, these parameters were further evaluated within the year. For both pH and GWC, no effect of production system was determined within either 2019 or 2020. (*P* > 0.05).

Ammonium concentration in the soil was unaffected by year but was affected by the interaction of production system and year, where a particularly high concentration of NH_4_^+^-N in the CT system at the 2019 sampling was observed, which was greater than all other combinations of system and year (*P* = 0.002, Fig. 1).

### Metatranscriptomic summary statistics and relative abundance

The total number of transcripts generated from each sample varied due to resequencing low-abundance samples at deeper depths. The total number of transcripts from each sample, total annotated transcripts, and percent of transcripts annotated are presented in Table 1.

**TABLE 1:** Total read pairs generated from Illumina NovaSeq 6000, read pair count after rRNA removal, percentage of non-rRNA read pairs, count of annotated read pairs, and percent of non-rRNA read pairs annotated per sample

Year	Conservation system^*a*^	Sample number	Total read pairs generated	Non-rRNA read pairs^*b*^	Percent non-rRNA read pairs	Annotated non-rRNA pairs^*c*^	Percent non-rRNA pairs annotated
2019	CT	Sample 2	7.23E+06	2.29E+05	3.2%	1.04E+05	45.4%
	CT	Sample 4	3.22E+07	1.01E+06	3.1%	4.68E+05	46.2%
	CT	Sample 9	6.57E+06	3.62E+05	5.5%	1.46E+05	40.3%
	NT	Sample 1	7.01E+06	4.25E+05	6.1%	1.44E+05	33.9%
	NT	Sample 5	5.66E+06	1.96E+05	3.5%	9.02E+04	46.1%
	NT	Sample 8	2.91E+07	1.11E+06	3.8%	5.34E+05	48.1%
	NTW	Sample 3	6.46E+06	2.09E+05	3.2%	8.76E+04	41.9%
	NTW	Sample 6	2.37E+07	9.57E+05	4.0%	3.73E+05	38.9%
	NTW	Sample 7	2.56E+07	8.84E+05	3.5%	3.72E+05	42.1%
2020	CT	Sample 11	5.17E+06	2.19E+05	4.2%	6.66E+04	30.4%
	CT	Sample 13	8.75E+06	2.37E+05	2.7%	7.18E+04	30.3%
	CT	Sample 18	8.76E+06	4.39E+05	5.0%	6.95E+04	15.8%
	NT	Sample 10	2.45E+07	1.06E+06	4.3%	3.44E+05	32.3%
	NT	Sample 14	6.39E+06	2.06E+05	3.2%	7.43E+04	36.0%
	NT	Sample 17	5.55E+06	2.19E+05	4.0%	1.74E+05	79.1%
	NTW	Sample 12	6.68E+06	2.25E+05	3.4%	7.12E+04	31.7%
	NTW	Sample 15	5.88E+06	1.62E+05	2.8%	5.37E+04	33.1%
	NTW	Sample 16	6.90E+06	1.52E+05	2.2%	5.80E+04	38.3%

^*a*^ Production system: CT, conventional tillage winter fallow; NT, no tillage winter fallow; NTW, no-tillage with a winter wheat cover crop.

^*b*^ Non-rRNA read pairs is the total number of reads for each sample after rRNA removal using ribodetector (24).

^*c*^ Annotated read non-rRNA pairs is the sum of the estimated counts generated using Kallisto (26).

**Fig 2:**
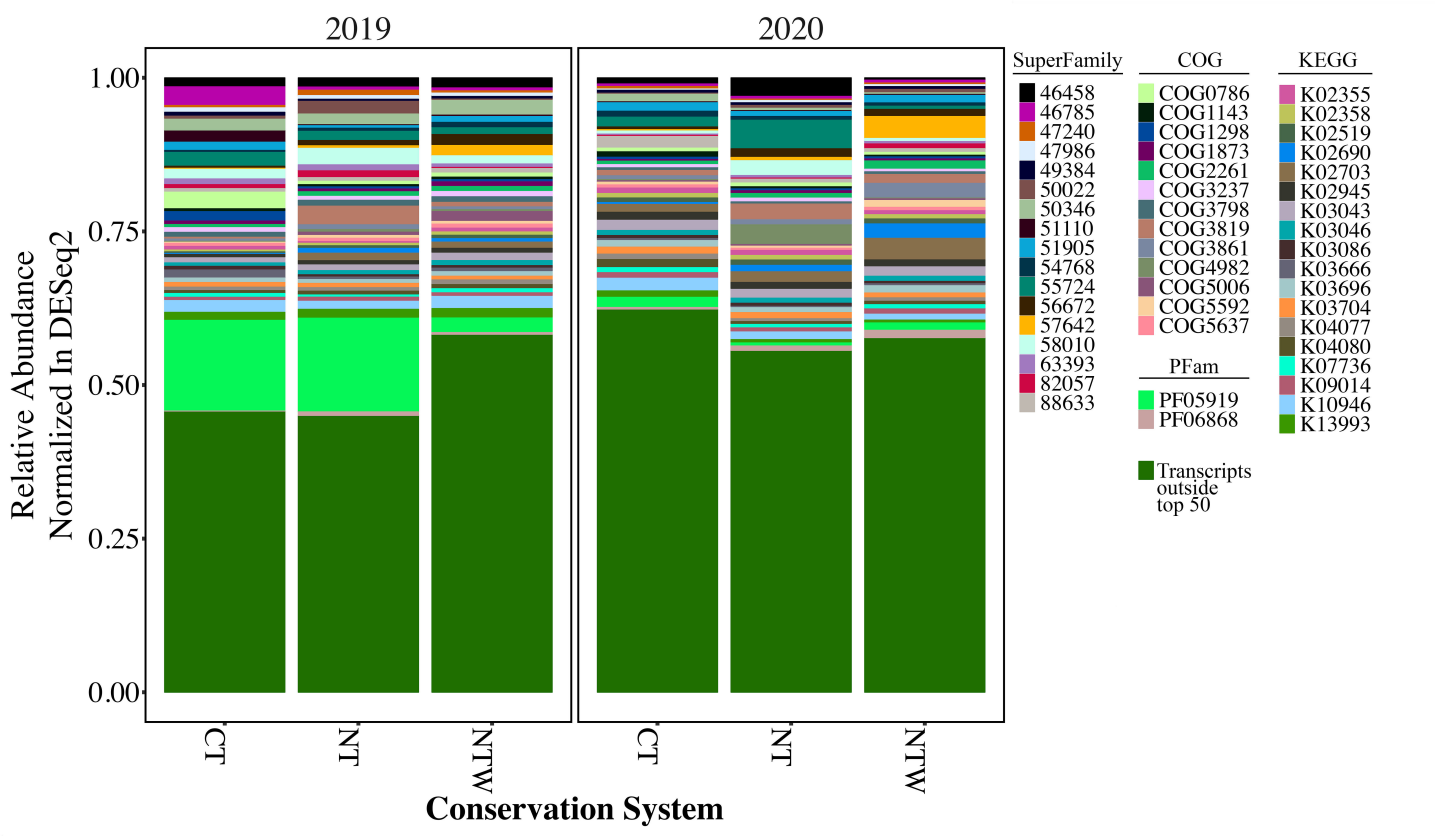
Relative abundance of the top 50 most abundant functional annotations and the sum of remaining functional annotations within production system and year. Relative abundance was calculated for each function as follows: dereplication of highly similar contigs, annotation of representative contigs, read mapping to dereplicated study-wide index, and finally normalized read counts produced by DESeq2 (27). CT, conventional tillage winter fallow; NT, no-tillage winter fallow; NTW, no-tillage with a winter wheat cover crop

Relative abundance of the top 50 most abundant functions is presented in Fig. 2. These highly abundant functions represented over 50% of the total annotated abundance for the CT and NT systems in 2019 and about 40% of total transcript abundance for the NTW system in 2019 and all production systems in 2020. Notably, this increase was related to the large abundance of the function annotated as PF05919, which corresponds to Mitovirus RNA-dependent RNA polymerase proteins and would not have been detected and removed by the filtering applied for bacterial rRNA transcripts. In 2019, this function represented roughly 15% of sequences in the CT system and 14% of sequences in the NT system compared with just 2% of sequences in the NTW system. In 2020, the prevalence of PF05919 was significantly reduced across all systems, representing less than 2% of sequences in each production system.

**Fig 3:**
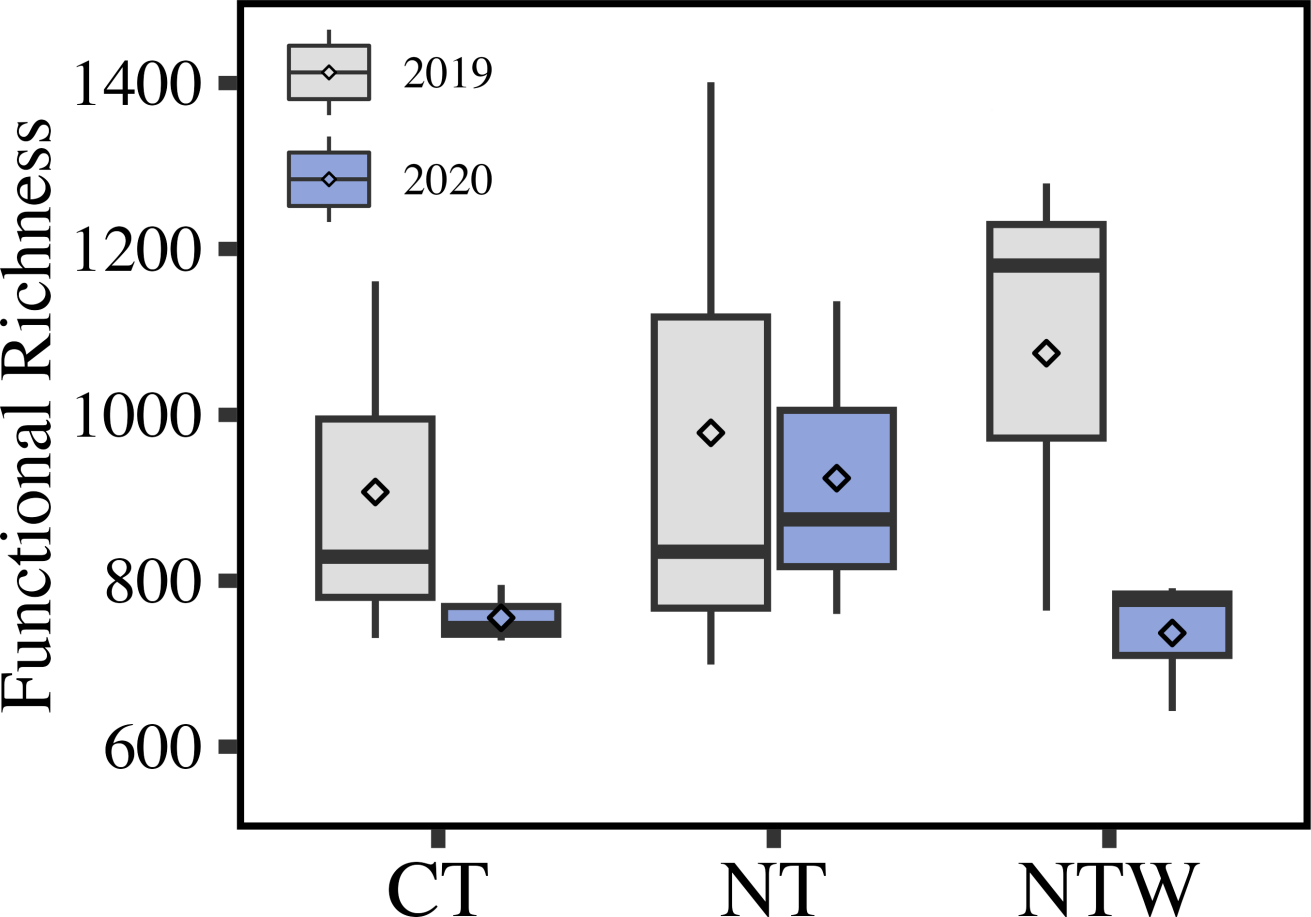
Richness of identified functions within production systems over the years. Richness was calculated as the number of unique functional annotations present in each year. CT, conventional tillage winter fallow; NT, no-tillage winter fallow; NTW, no-tillage with a winter wheat cover crop.

Richness, abundance of each functional annotation, was not significantly different between years, production systems, or their interaction (year × production system, *P* > 0.05; Fig. 3). In general, greater richness was observed in 2019 (987 functions) compared with 2020 (805 functions) across production systems. A weak trend of increasing functional richness from the CT (907 functions) to NTW system (1074 functions) occurred in 2019. However, the NT system (924 functions) had generally greater functional richness than the CT (755 functions) and NTW systems (737 functions) in 2020.

### Ordination of gene expression profiles

**Fig 4:**
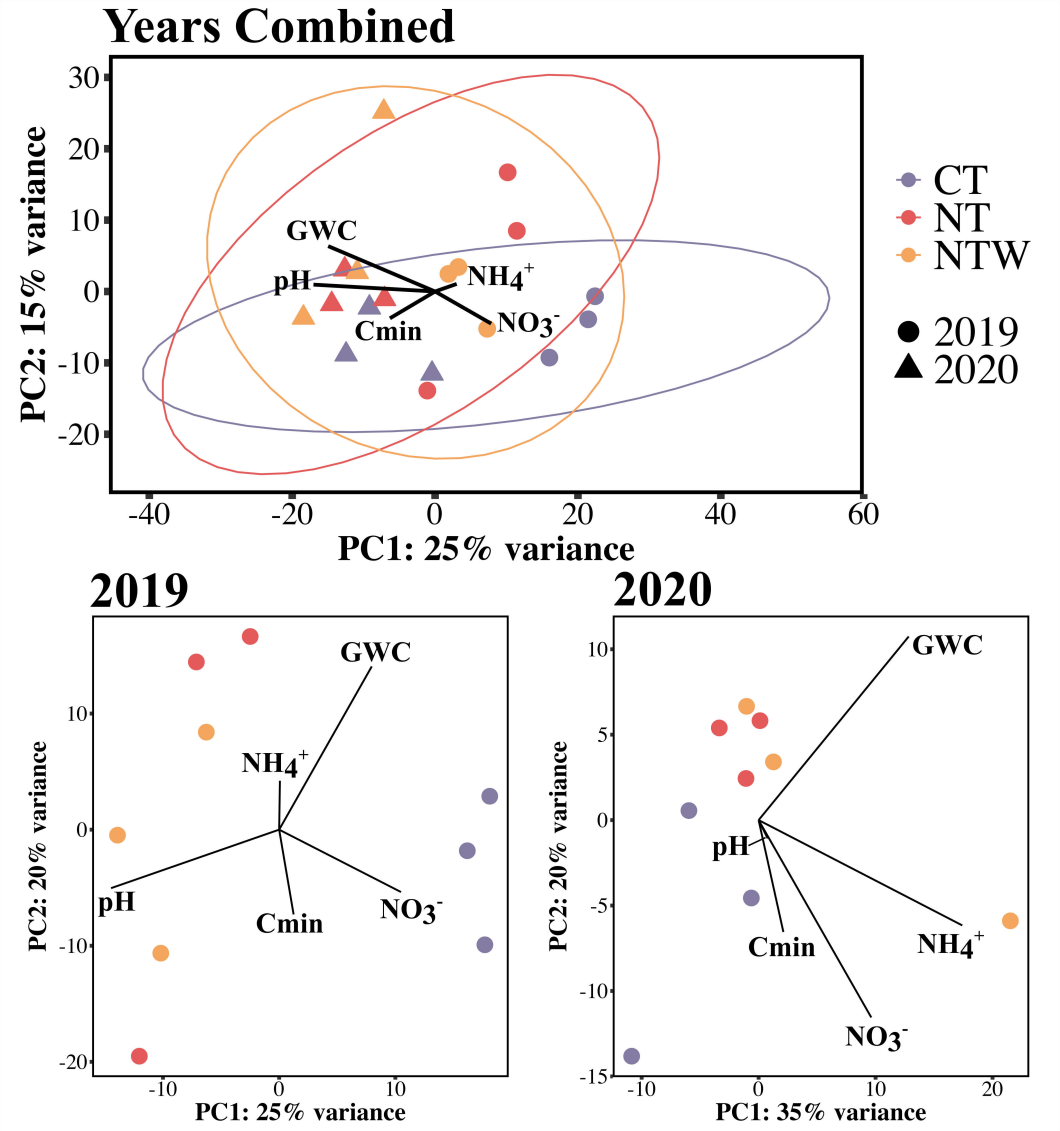
Principal components analyses of microbial functional expression within production systems across years and within each year of the study. Ellipses represent 95% confidence intervals. Vectors fit to the PCAs represent the relationships between soil parameters and the dissimilarity of gene expression between samples. CT, conventional tillage winter fallow; NT, no-tillage winter fallow; NTW, no-tillage with a winter wheat cover crop

Principal components analysis of the differential expression profiles generated with DESeq2 revealed slight clustering of production systems across both years (Fig. 4). In general, gene expression in the CT system was slightly different than that of the NT and NTW systems across years and was associated with PC2, while sampling year was associated with PC1 (Fig. 4). Using PERMANOVA, it was determined that functional expression was affected by both production system (*P* = 0.036) and year (*P* ≤ 0.001) individually but not their interaction (*P* = 0.391). Gene expression differed between years when analyzed using pairwise comparison (*P* ≤ 0.001); however, only the CT and NTW systems weakly differed in expression (*P* = 0.090; adj. *P* = 0.270).

Environmental factors (Cmin, NH_4_^+^-N, NO_3_-N, pH, GWC) were fit to the ordination with GWC (r^2^ = 0.66, *P* ≤ 0.001), pH (r^2^ = 0.74, *P* ≤ 0.001), NH_4_^+^-N concentration (r^2^ = 0.33, *P* = 0.034) being significantly associated with functional expression across the samples. When these parameters were then correlated with each PC, all three parameters were significantly correlated (*P* < 0.05) with PC1 (GWC, r = −0.77; pH, r = −0.86; NH_4_^+^-N, r = 0.57), and no parameters were correlated with PC2. These relationships between functional expression and soil characteristics generally correspond to the yearly differences in the soil characteristics observed in Fig. 1.

### Ordination of expression within years

Because of the clear effect of year on gene expression in Fig. 4, PCA was also conducted within year (Fig. 4), which further revealed the difference between the CT and non-tilled systems (NT and NTW), where the clustering was driven by both PCs. In both 2019 (PERMANOVA *P* = 0.026) and 2020 (PERMANOVA *P* = 0.021), functional expression was affected by production system. In pairwise comparisons, no difference was determined between production systems in either year (*P* > 0.05).

In 2019, only GWC (r^2^ = 0.65, *P* = 0.034) and NH_4_^+^-N concentration (r^2^ = 0.87, *P* = 0.006) were significantly associated with functional expression. When soil characteristics were correlated with the PCs directly, NH_4_^+^-N concentration was positively correlated with PC1 (r = 0.89), while GWC was positively correlated with PC2 (r = 0.68). In addition, soil pH was negatively correlated (*P* = 0.025, r = −0.73) with PC1 in the direct correlation analysis for the 2019 samples. In 2020, only GWC was significantly associated with the ordination of functional expression (r^2^ = 0.70, *P* = 0.013). However, both GWC (r = 0.70) and NH_4_^+^-N concentration (r = 0.89) were positively correlated with PC1 in the direct correlation.

### Differential expression

**Fig 5:**
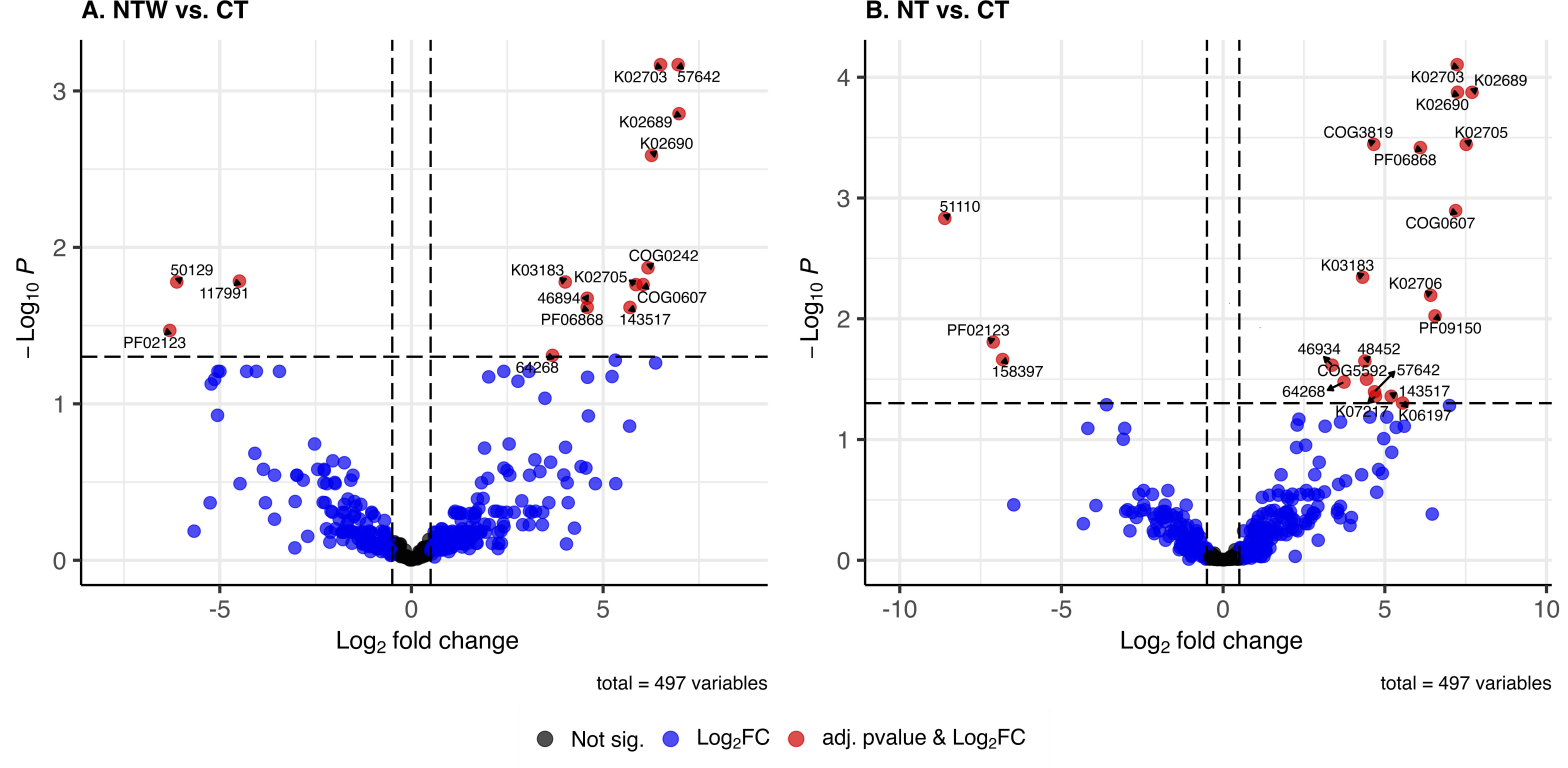
Volcano plot of functions significantly differentially expressed between (A) no-tillage with winter wheat cover (NTW) and conventional tillage systems (CT) and (B) no-tillage winter fallow (NT) and CT systems. Functions with a log fold change greater than 0.5 are highlighted in blue, and functions with both a log fold change greater than 0.5 and an adjusted *P* < 0.05 are highlighted in red. Descriptions of significantly differentially expressed functions are provided in Table 2.

Differential expression at the function level revealed the enrichment of several functions between the NTW and CT systems and the NT and CT systems across years (adjusted *P* < 0.05; Fig. 5; Table 2). No function was differentially expressed between the NT and NTW systems. There were 15 differentially expressed functions between the NTW and CT systems (Fig. 5) across the years, of which only three functions (PF02123, 50129, and 11799) were enriched in the CT system compared with the NTW system (Table 2). Notably, several functions upregulated for the NTW system were related to photosynthesis (K02705, K02690, K02703, and K02689). Twenty (20) functions were differentially expressed between the NT and CT systems across the years (Fig. 5), with only three functions (51110, PF02123, and 158397) being enriched in the CT system compared with the NT system (Table 2). Similar to the NTW system, several functions related to photosynthesis were upregulated in the NT system (K02706, K02703, K02690, K02705, and K02689). These photosynthetic genes were taxonomically identified to belong to several species of cyanobacterial organisms (Table S4).

**TABLE 2:** Differentially expressed functional annotations between production systems across both study years^*c*^

Comparison^*a*^	Database ID^*b*^	Description	log2 fold change	*P*-value	Adjusted *P*-value
NTW vs. CT	PF02123	Viral RNA-directed RNA-polymerase	−6.304	0.001	0.034
	50129	GroES-like superfamily	−6.122	<0.001	0.017
	117991	YbeD/HP0495-like	−4.488	<0.001	0.016
	64268	Phosphatidylinositol phosphate binding	3.680	0.001	0.049
	K03183	Demethylmenaquinone methyltransferase/2-methoxy-6-polyprenyl-1,4-benzoquinol methylase	4.013	<0.001	0.017
	46894	C-terminal effector domain of the bipartite response regulators	4.584	<0.001	0.021
	PF06868	Protein of unknown function (DUF1257)	4.585	0.001	0.024
	143517	TRCF domain-like	5.701	0.001	0.024
	K02705	Photosystem II CP43 chlorophyll apoprotein	5.857	<0.001	0.017
	COG0607	Rhodanese-related sulfurtransferase	6.044	<0.001	0.017
	COG0242	Peptide deformylase	6.171	<0.001	0.013
	K02690	Photosystem I P700 chlorophyll a apoprotein A2	6.264	<0.001	0.003
	K02703	Photosystem II P680 reaction center D1 protein	6.498	<0.001	0.001
	57642	Cholecystokinin A receptor, N-domain	6.957	<0.001	0.001
	K02689	Photosystem I P700 chlorophyll a apoprotein A1	6.981	<0.001	0.001
NT vs. CT	51110	Alpha-D-mannose-specific plant lectins	−8.607	<0.001	0.001
	PF02123	Viral RNA-directed RNA-polymerase	−7.109	<0.001	0.016
	158397	TM1646-like domain superfamily	−6.819	0.001	0.022
	46934	UBA-like superfamily	3.369	0.001	0.024
	64268	Phosphatidylinositol phosphate binding	3.739	0.001	0.033
	K03183	Demethylmenaquinone methyltransferase / 2-methoxy-6-polyprenyl-1,4-benzoquinol methylase	4.312	<0.001	0.005
	48452	P-P-bond-hydrolysis-driven transmembrane transporter activity	4.383	0.001	0.022
	COG5592	Hemerythrin domain-containing protein	4.435	0.001	0.032
	COG3819	Uncharacterized membrane protein	4.659	<0.001	<0.001
	57642	Cholecystokinin A receptor, N-domain	4.681	0.002	0.040
	K07217	Manganese catalase	4.710	0.002	0.044
	143517	TRCF domain-like	5.199	0.002	0.044
	K06197	Cation transport regulator	5.550	0.002	0.050
	PF06868	Protein of unknown function (DUF1257)	6.099	<0.001	<0.001
	K02706	Photosystem II P680 reaction center D2 protein	6.417	<0.001	0.006
	PF09150	Orange carotenoid protein, N-terminal (Photoprotection)	6.552	<0.001	0.009
	COG0607	Rhodanese-related sulfurtransferase	7.190	<0.001	0.001
	K02703	Photosystem II P680 reaction center D1 protein	7.236	<0.001	<0.001
	K02690	Photosystem I P700 chlorophyll a apoprotein A2	7.248	<0.001	<0.001
	K02705	Photosystem II CP43 chlorophyll apoprotein	7.516	<0.001	<0.001
	K02689	Photosystem I P700 chlorophyll a apoprotein A1	7.697	<0.001	<0.001

^*a*^ Comparison: CT, conventional tillage, winter fallow; NT, no-tillage winter fallow; NTW, no-tillage winter fallow with a winter wheat cover crop.

^*b*^ Database ID corresponds to one of the five databases used for annotation. IDs without a preceding letter (i.e., 51445) correspond to SuperFamily annotations. Other IDs: K, KEGG; COG, COG; PF, PFam.

^*c*^ Comparisons with adjusted *P* < 0.05 are presented.

Despite an observed increase in PF05919 with relative abundance, the function was not differently expressed between the NTW and CT (*P* = 0.032; adj. *P* = 0.29) or NTW and CT systems (*P* = 0.026; adj. *P* = 0.99). However, a separate viral function (PF02123) associated with plant/fungal viruses was enriched in the CT and NT systems compared with the NTW system. Differential expression between years was more robust than between production systems, with 58 differentially expressed functions between years, across production systems. Of these, 15 were enriched in 2020 compared with 43 enriched in 2019 (Table S5). Interestingly, an oxidoreductase associated with nitrate reduction was enriched in 2019 compared with 2019 (SuperFam 50022).

## DISCUSSION

Semi-arid croplands are expected to expand under global climate change predictions (37). Understanding the impact conservation management can have on microbially driven biogeochemical processes in semi-arid soils will be vital for making management decisions to maintain economic and environmental sustainability of semi-arid agronomic production. After 5 years of no-tillage implementation, gene expression differed among the levels of conservation management and can be generally delineated between the tilled (CT) and non-tilled systems (NT, NTW).

### Change in function does not directly parallel change in structure

No significant change in overall functional richness was observed between any production system, nor was richness different across study years. Thus, we reject our hypothesis that due to previously observed decreases in diversity between the NTW and non-cover cropped systems (10) we would observe a corresponding decrease in unique functions for the NTW system. In contrast, expression profiles in the CT system were generally different than both the non-tilled systems, suggesting that the change in soil environment introduced with the removal of tillage is altering microbial community function, rejecting our hypothesis that the NTW system would produce a more “unique” gene expression profile.

It is well known that changes in microbial community structure do not always confer changes in function (11), and the strength of environmental selection that was apparent in the overall low diversity associated with community structure likely has a similarly strong effect on the functional expression in this system. However, the differences in functional expression between systems were not strongly associated with any measured soil parameter, suggesting that an additional soil physicochemical parameter conferred the difference in expression. A caveat to this assumption is that there was a clear pattern in functional expression between years, which was strongly associated with the observed yearly changes in soil pH and GWC. The effect of year and yearly variable soil parameters further supports the strength of the environmental filter on microbial function in these soils. Despite the correlation between GWC and yearly shifts in functional expression, there is no apparent environmentally relevant reason for these differences. The pH observed in 2020 for these samples is more similar to the other years of the study (17) and thus these specific samples from 2019 appear to be an anomaly (although consistent across the growing season). Because of this, our interpretations are limited when observing across years. In regard to GWC, both systems received rain or precipitation within the previous 2 weeks of sampling as noted above, so the major discrepancy between years should not be attributable to a simple difference in management immediately prior to sampling.

Due to the clear effect of year on bacterial gene expression (associated with changes in soil pH and GWC), gene expression was examined within year. The similarity of expression between the non-tilled systems compared with the CT system was more apparent within each year than across the 2 years together. In 2019, this difference was strongly correlated to the high concentration of NH_4_^+^-N in the CT system compared with the non-tilled systems, while in 2020, GWC was strongly correlated with functional expression. In both cases, both the management of the soil and the environment likely played a role in altering functional expression within the non-tilled systems compared with the tilled systems. However, like the across-years analyses, the relationships between soil physicochemistry and gene expression do not necessarily explain the observed dissimilarity between tilled and non-tilled systems.

### Examining function-specific differences between levels of conservation management

Because the relationship between soil parameters and gene expression remained inconclusive, we closely examined specific changes in functional capabilities to determine what was driving differences in our bacterial gene expression profiles. A notable example of this occurred in 2019, where the prevalence of the function PF05919 (Mitovirus RNA-dependent RNA polymerase protein) was greater in the non-cover cropped systems (CT, NT) compared with the cover cropped system (NTW). Mitoviruses have been reported to infect soil fungi (38), including fungal pathogens, such as *Fusarium oxysporum* (39), which causes Fusarium wilt in cotton (40). The identification of this viral function does not directly indicate a Fusarium wilt outbreak in the CT and NT systems or a specific reduction in disease incidence in the NTW system in 2019, but cursory analyses of cotton lint yield in 2019 reveal a strong yield reduction for those systems compared with the NTW system (*data not shown*). This trend did not continue in 2020; however, PF05919 was similarly abundant across all systems, but a similar pattern in cotton lint yield was observed (*data not shown*). Nevertheless, the potential for the NTW system to suppress disease aligns with previous results in long-term no-tillage systems (41) and further indicates the potential for cover crops to increase sustainability in semi-arid soils.

The broader changes in functional expression between tilled and non-tilled systems were associated with significant changes in several functions associated with photosynthetic processes. These functions were enriched for both non-tilled systems, suggesting that tillage can reduce the prevalence and activity of photosynthetic organisms, likely through burial of these organisms with tillage. These organisms were taxonomically identified as cyanobacteria, and specifically several species that are reported to form biocrusts in arid ecosystems (42). Biocrusts (biological soil crusts) are biological structures developed by combinations of soil organisms, such as cyanobacteria, microalgae, fungi, and others that conduct key ecological functions in arid and semi-arid ecosystems (24). The timeline for the formation of biocrusts is plausible in these soils as crusts can develop within 2 years following the removal of disturbance—i.e., tillage (43). However, the prevalence of cyanobacteria suggests that these crusts are in the early stages of development (24), which may be the extent of crust formation due to other less severe disturbance sources, such as field vehicle and foot traffic consistently setting back crust development. As further evidence of the presence and activity of these biocrusts, PF09150 (Orange carotenoid protein, N-terminal) was significantly more expressed in the NT system compared with the CT system. This protein is responsible for high light tolerance in cyanobacteria (44), which would be advantageous in the bare soil of the NT system, and likely less necessary for the NTW system due to increased shading with the cover crop residue.

The increase in biocrust-associated transcripts in both the NT and NTW treatments provides strong evidence that implementation of the conservation practices (i.e., no-tillage) is resulting in soil microbiomes more ecologically similar to the natural ecosystem. While no-tillage and cover cropping are aimed at creating a “healthier” soil ecosystem, the increase in biocrust-associated transcripts suggests there is an apparent harshness to the surface of no-tillage systems compared with tilled semi-arid soil and that organisms specialized for survival in these conditions have a fitness advantage in these systems. In other words, the organisms on the surface of semi-arid no-tillage systems thus must adapt, or be specially adapted for, conditions much more similar to the natural arid and semi-arid ecosystems that surround the managed soils of the Southern High Plains.

### Gene expression differed by year

In addition to some trends in gene expression observed between production systems, functional expression was distinct between years, with over 50 upregulated or downregulated functions between the two field seasons. Notably, functions associated with two-component systems, nitrate reductase, and heat-shock proteins were upregulated in 2019 compared with 2020. When considering that the driving factors behind the expression profiles of each year, pH and GWC, were increased in 2020 compared with 2019, it is reasonable that increases in stress-response genes would be observed in 2019.

### Conclusions

Soil microbial gene expression in semi-arid agricultural soils is likely regulated by numerous, complex soil physicochemical interactions that may be altered with the implementation of conservation management strategies, such as no-tillage and cover cropping. This is apparent in the yearly differences in functional expression profiles, which trend strongly with soil pH and water content but are not strongly associated with observed similarities within each production system. However, the removal of tillage and growth of a cover crop did induce a change in the functional expression profile of the microbial community compared with conventionally tilled soil that may not be associated with the soil parameters measured for this study. First, a notable reduction in the prevalence of a potential viral indicator of crop fungal infection was observed for the NTW system in the first year of metatranscriptomic analysis compared with the non-cover cropped systems. Whether this potential infection was inhibited by the NTW system or promoted by the non-cover cropped systems is beyond the scope of this study but suggests that the benefits of cover cropping in semi-arid soils may extend beyond soil stabilization, water holding, and carbon inputs. Second, the removal of tillage in the NT and NTW systems corresponds with a large increase in the expression of photosystem genes associated with biocrust-forming cyanobacteria. The formation of biocrusts in semi-arid agricultural soils is understudied, but the potential benefits their formation offer to the soil ecosystem should be considered in future management planning and research. The findings of this study reflect the potential to directly alter microbial function in semi-arid cropland soils through common soil management practices, although the specific drivers of these alterations do not seem to be related to commonly measured soil parameters. Furthermore, the observed changes in bacterial gene expression appear to provide unexpected benefits to the environmental and agronomic sustainability of these crop production systems that may be useful under future climate change scenarios.

## Data Availability

The raw RNA sequence data (and relevant soil metadata) generated and analyzed for this current study are available in the NCBI Sequence Read Archive (SRA) repository under BioProject no. PRJNA1128048.

## SUPPLEMENTAL MATERIAL

Supplemental material aem.00184-25-s0001.pdf Results from the second differential expression approach noted in the paper; Tables S2 and S3.

Table S1 aem.00184-25-s0002.xlsx Full results of differential expression analysis at the contig level.

Table S4 aem.00184-25-s0003.xlsx Taxonomic identifications of the differentially expressed photosystem genes.

Table S5 aem.00184-25-s0004.xlsx Differential expression analysis results between study years.

ASM does not own the copyrights to Supplemental Material that may be linked to, or accessed through, an article. The authors have granted ASM a non-exclusive, world-wide license to publish the Supplemental Material files. Please contact the corresponding author directly for reuse.
