# Supplementary material for: No-tillage systems promote bacterial photosynthetic gene expression in low carbon, semi-arid surface soils
Source: Appl Environ Microbiol. 2025 Mar 10;91(4):e00184-25. doi: 10.1128/aem.00184-25 (PMC12016546; doi:10.1128/aem.00184-25)
Supplement: Supplemental material — Results from the second differential expression approach noted in the paper; Tables S2 and S3. [file aem.00184-25-s0001.pdf]

SUPPLEMENTARY MATERIAL

Transcript-Level Analysis of Gene Expression

Ordination of Transcript Expression Profiles

Principal components analysis of the transcript-level differential expression profile generated with DESeq2 revealed clustering of sampling years along PC1 and clustering of conservation systems along PC2 (Supplemental Figure 1). Similar to the function-level analysis presented in the main text, the strong effect of year on gene expression was further investigated for within-year effects of conservation system (Supplemental Figure 1). Within each year, the difference in expression profile between the CT system and the two non-tilled systems (NT and NTW) was more apparent and occurred along both PC1 and PC2 (Supplemental Figure 1).

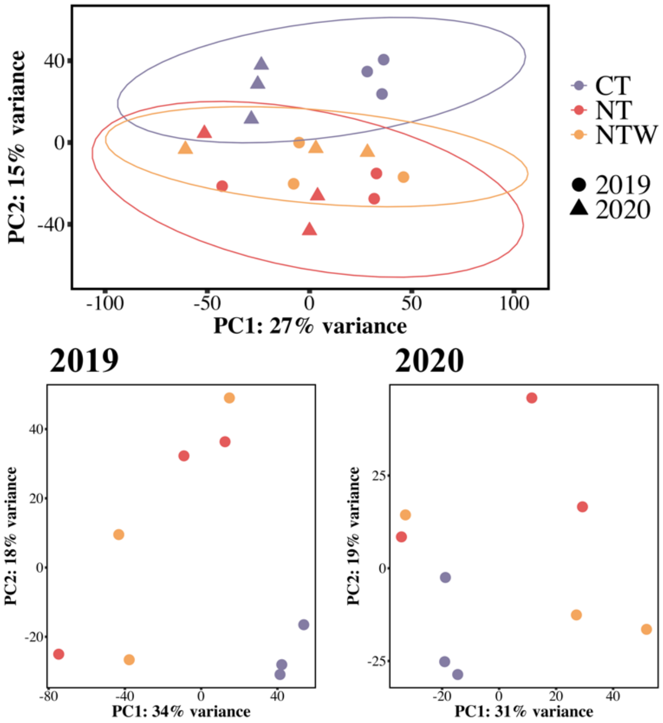

Supplemental Figure 1. Principal components analyses of transcript expression within conservation system across years and within each year of the study. Ellipses represent 95% confidence intervals. CT, conventional tillage winter fallow; NT, no-tillage winter fallow; NTW, no-tillage with a winter wheat cover crop.

Environmental factors (Cmin, NH_4^+-N, NO_3^--N, pH, GWC) were fit to the ordinations where for the full analysis (Conservation system × Year) soil pH (r^2=0.54; p=0.002) and NO_3^--N concentration (r^2=0.26; p=0.035) were significantly associated with the ordination. However, when these environmental factors were correlated with the PCs, only pH was correlated with the axes, where pH was significantly correlated with PC1 (p=0.001; r=-0.79). In 2019, GWC

(r^2=0.89; p=0.005) and soil pH (r^2=0.75; p=0.017) were significantly associated with the ordination. In addition, both parameters were significantly correlated with PC1 of the 2019 ordination (GWC: p=0.015, r=0.77: pH: p=0.003, r=-0.86). In 2020, no environmental factors were significantly associated with the ordination. However, both GWC (p=0.046; r=0.68) and NH_4^+-N concentration (p=0.038; r=0.69) were correlated with PC1 in 2020.

Differential Expression of Transcripts Between Conservation Systems

A large number of transcripts were differentially expressed between the CT system and the NTW and NT systems (Supplementary Table 1). Specifically, between the CT and NTW systems 187 transcripts were expressed more in the NTW system while 57 were more expressed in the CT system. In addition, there were 85 outliers of expression and 511 transcripts classified as "low counts" for the CT and NTW comparison. Between the CT and NT systems, 492 transcripts were expressed more in the NT system and 67 were expressed more in the CT system, 85 were classified as outliers and 1023 were classified as "low counts". Unlike the lack of difference in differential expression between NTW and NT at the function level, 3 transcripts were expressed more for the NTW system while 9 were expressed more for the NT system. Again, 85 transcripts were classified as outliers for this comparison and 2860 were classified as "low counts".

Supplementary Tables

Supplementary Table 1 is attached separately as a Microsoft Excel File due to the number of transcripts differentially expressed between conservation systems. We must note that not all transcripts differentially expressed are attributed to a functional annotation by the databases used in the NMDC EDGE Metagenomic Annotation Workflow.

Supplementary Table 2. Repeated-measures analysis of variance (Two-way ANOVA) table of soil chemical characteristics.

Environmental Factor[1]	Analysis of Variance (p-values)		
	Con. Sys.[2]	Year	C×Y[3]
pH	0.356	**0.002**	0.357
GWC	0.112	**0.001**	0.112
Cmin	0.877	0.461	0.877
NH_4^+-N	**0.002**	0.094	**0.002**
NO_3^--N	0.832	0.149	0.832

[1] Environmental Factors include soil pH, gravimetric water content (GWC), mineralizable carbon content (Cmin), ammonium concentration (NH_4^+-N), and nitrate concentration (NO_3^--N)

[2] Con. Sys., conservation system main factor comparison (conventional tillage vs. no-tillage vs. no-tillage with winter wheat cover crop)

[3] C×Y, interaction term of conservation system and year

Supplementary Table 3. Mean soil characteristic values within conservation system and year.

Year	Con. Sys.[1]	pH[2]	GWC	Cmin	NH_4^+	NO_3^-
			g water g^{-1} soil	mg C kg^{-1} soil	mg N kg^{-1} soil	
2019	CT	6.13	8.38	60.1	19.44*	10.82
	NT	6.84	7.83	45.7	4.09	4.62
	NTW	6.80	7.90	63.7	5.84	11.77
2020	CT	7.77	11.92	70.0	6.03	4.47
	NT	7.83	12.86	64.5	6.18	2.11
	NTW	7.95	13.74	75.7	8.12	6.50

* Significantly different at $p<0.05$, ANOVA

[1] Con. Sys: Conservation system; CT, conventional tillage winter fallow; NT, no-tillage winter fallow; NTW, no-tillage with winter wheat cover crop

[2] pH, soil pH (1:2 soil-water dilution); GWC, gravimetric water content (7 days drying at 60°C); Cmin, mineralization carbon content (3-day rewetting CO_2 flush); NH_4^+, ammonium concentration (KCl extract); NO_3^-; nitrate concentration (KCl extract)

Supplementary Table 4. Taxonomic annotations of photosystem functions identified to be differentially expressed between the tilled (CT) and no-tillage (NT and NTW) systems.
(See Separate Excel Worksheet File)
